# O-20 - IDENTIFYING HIGHER RISK COMMUNITY NURSING PATIENTS FOR TARGETED SCREENING DURING AN OUTBREAK OF INVASIVE GROUP A STREPTOCOCCUS (GAS) EMM 89.0

**DOI:** 10.1093/pubmed/fdag046.017

**Published:** 2026-07-09

**Authors:** Ms Rebecca Hams, John Harle, Iain Roddick, Ade Sorungbe, Steve Dolan, Clare Johnstone, Jennifer Day, Sue Devenish, Emma Paine, Smita Kapadia, Theresa Lamagni, Victoria Kirkby

**Affiliations:** UK Health Security Agency; Central London Community Healthcare NHS Trust; UK Health Security Agency; UK Health Security Agency; Central London Community Healthcare NHS Trust; Central London Community Healthcare NHS Trust; Hertfordshire and West Essex Integrated Care Board; Hertfordshire and West Essex Integrated Care Board; UK Health Security Agency; UK Health Security Agency; UK Health Security Agency; UK Health Security Agency

## Abstract

**Background:**

Between January and June 2025, nine invasive group A streptococcal (iGAS) emm89.0 cases were identified across six care homes and one household, all receiving wound care from the same community nursing team. One genetically linked asymptomatic staff member was also detected. Full caseload screening was not feasible, and limited published guidance on targeted screening highlighted the need for rapid, operational approaches

**Objectives:**

To create a reproducible approach for identifying higher-risk patients for targeted screening.

**Methods:**

Two datasets from community nursing records were reviewed to identify patients with contact with the staff member. First, for outbreak settings (care homes with a case), we identified residents who had received at least two visits in the six months preceding the first confirmed case. Second, for non-outbreak settings, we identified individuals visited in the 28 days before the staff member’s positive sample. Both datasets were cross-checked with outbreak linelists and national laboratory data. Eligible individuals’ clinical records were reviewed to determine ongoing care and suitability for screening. Screening involved throat and /or wound swabs.

**Results:**

One hundred and two individuals were assessed. Fourteen met criteria for targeted screening: nine in outbreak settings and five in non-outbreak settings. Of these, one had died, one was no longer eligible and four declined. Eight individuals were screened, all tested negative on throat and/or wound swabs. No asymptomatic carriers were identified among residents screened.

**Conclusions:**

Targeted screening provides a feasible alternative to mass screening in community-associated GAS outbreaks when resources are limited. Although no asymptomatic cases were detected, the approach demonstrated a structured, reproducible method for prioritising patients. A key challenge was gaining consent from relatives where patients lacked capacity, highlighting the need for clearer processes and further work in this area. Further validation would help assess the method’s sensitivity and refine its use in future outbreaks.

